# DNAism: exploring genomic datasets on the web with Horizon Charts

**DOI:** 10.1186/s12859-016-0891-2

**Published:** 2016-01-27

**Authors:** David Rio Deiros, Richard A. Gibbs, Jeffrey Rogers

**Affiliations:** Human Genome Sequencing Center, Baylor College of Medicine, One Baylor Plaza, Houston, USA; Department of Molecular and Human Genetics, Baylor College of Medicine, One Baylor Plaza, Houston, USA

**Keywords:** Bioinformatics, Genomics, Sequencing, JavaScript, Web, Visualization

## Abstract

**Background:**

Computational biologists daily face the need to explore massive amounts of genomic data. New visualization techniques can help researchers navigate and understand these big data. Horizon Charts are a relatively new visualization method that, under the right circumstances, maximizes data density without losing graphical perception.

**Results:**

Horizon Charts have been successfully applied to understand multi-metric time series data. We have adapted an existing JavaScript library (Cubism) that implements Horizon Charts for the time series domain so that it works effectively with genomic datasets. We call this new library DNAism.

**Conclusions:**

Horizon Charts can be an effective visual tool to explore complex and large genomic datasets. Researchers can use our library to leverage these techniques to extract additional insights from their own datasets.

**Electronic supplementary material:**

The online version of this article (doi:10.1186/s12859-016-0891-2) contains supplementary material, which is available to authorized users.

## Background

Sharing and communicating about large and intricate datasets produced by high throughput sequencing can be a challenging task. Visual channels are an effective way to explore data. However, the accelerating increase in data quantity is pushing the limits of current approaches for representing these datasets visually without sacrificing accuracy or graphical perception. Overall data volume is growing: both the amount of data per study and the number of subjects. Thus, more effective visualization techniques are needed to understand the most challenging genomic sequencing datasets.

Horizon Charts [1, 2] have proved to be an effective [3] visualization approach when working with multi-metric time series encoded data. In high throughput sequencing, BED format files are extremely widely-used for capturing values associated with genomic coordinates. In time series, metrics are monitored over time, however, BED files use genomic coordinates. We have adapted a time series JavaScript library to the genomic domain. We call our new library DNAism.

### State of the art

**Fig. 1:**
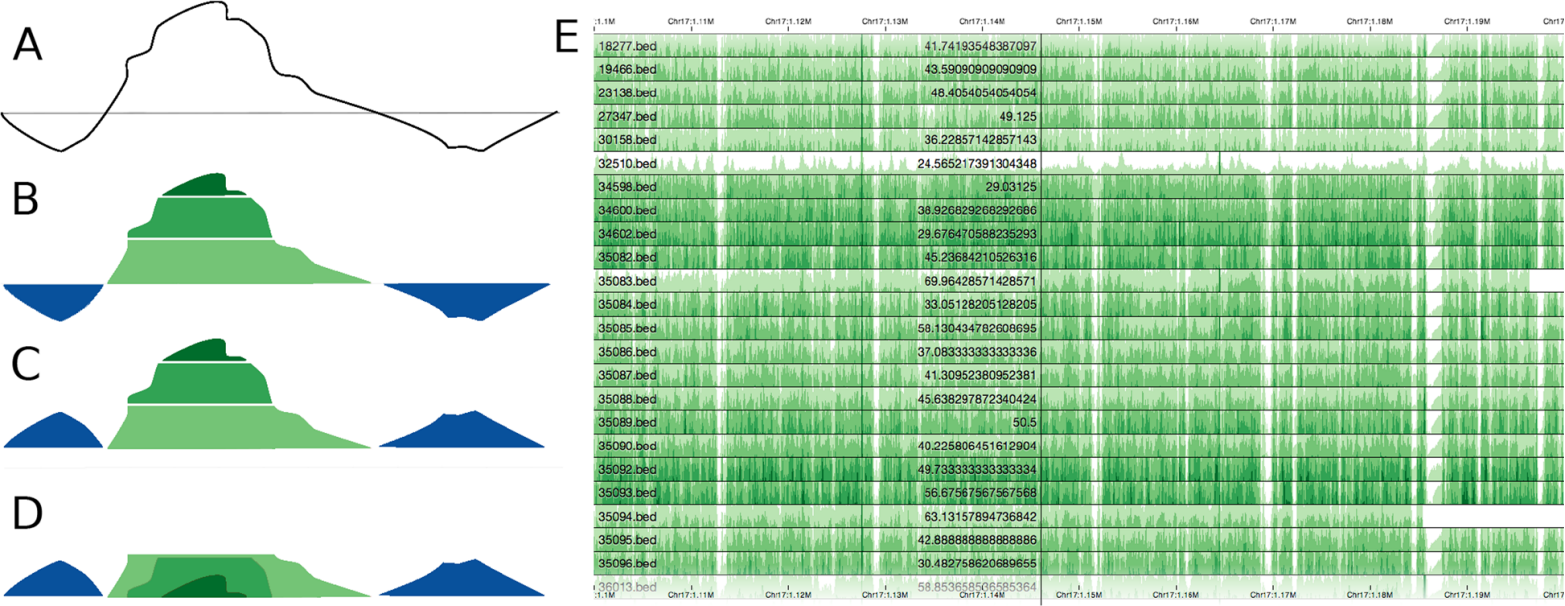
Horizon Charts emerge from applying a set of changes to traditional line graphs (**a**). We start by coloring the underlying area of the line graph, using different hues for positive and negative values. Next, we divide the graph into bands and apply a gradient of color that increases along with the quantitative value of the variable we are investigating (**b**). In the next step, negative values are flipped over the baseline (**c**), effectively reducing the required vertical space by two fold. In a final step, bands are collapsed making all of them start at the baseline and providing another level of space reduction (**d**). We used this technique to rapidly identify problematic samples when performing quality control on large scale sequencing results. You can see the read depth across whole genome sequences from 24 rhesus macaque samples (30x coverage) for genomic region Chr17:1.1M-1.2M (**e**). There are regions consistently underrepresented across all the samples and sample 32510 has low coverage across the whole genomic region. Note that the variable we are exploring in this example, read depth, does not contain negative values. Therefore, only green hues appear in (**e**)

We are not aware of any implementation of Horizon Charts within the domain of genomics research or clinical genetics. In the context of web-based genome browsers [4, 5], these tools use traditional line charts (http://goo.gl/KRTmsJ) to visually represent the variables of interest [Additional file 1: Figure S1]. For cases (rather common) in which the number of variables we are interested in is high, the effectiveness of this traditional mechanism declines significantly, forcing the user to load or display multiple tracks on different pages. Horizon Charts will help researchers deal with this common task and scenario, as the visual technique minimizes the visual space necessary to view large amounts of data without losing graphical perception Fig. 1.

Future development and enhancement of genome browsers should see Horizon Charts as one flexible and efficient answer to the challenges faced when displaying large amounts of data. In the appropriate circumstances, this approach will provide significant benefits to browser developers. Greater effectiveness in the display of data will in turn help researchers explore that information more efficiently and conveniently.

## Implementation

Contrary to time series data, in genomic datasets, the variable under study (x-axis) is associated with chromosomal coordinates instead of timestamps. We have modified an existing time series data visualization library (based on D3 [6]) called Cubism to support genome coordinate data. This makes DNAism a flexible and effective tool to explore multi-sample genomic datasets using Horizon Charts.

To visualize genomic datasets, we have modified most of the software components of the original Cubism library (http://drio.github.io/dnaism). The two major components are ‘context’ and ‘source’. The ‘context’ component performs several functions. Most importantly, it defines the region of the genome we want to explore. This component also specifies, in pixels, how much vertical space we have available for the visualization. The ‘source’ component parses the genomic raw data and generates the data points necessary for visualization. Our library provides two sources: ‘bedfile’ and ‘bedserver’. Once the sources are created we can use the metric component to instantiate metrics associated with specific samples. Finally, the horizon component encapsulates the functionality necessary to create the visual elements.

One of the crucial features of DNAism is the ability to efficiently parse and load the genomic data for visualization. We have provided two alternatives via the bedfile and the bedserver sources. A bedfile is a simple solution that loads all the genomic information in memory and returns the relevant data when queried. However, this approach is not adequate for larger datasets, especially those involving multi-sample data. To handle such cases, the bedserver source can be used. A bedserver is a dedicated server that implements a RESTful API interface. The client’s code running in the browser can send queries to this server to obtain the data of interest. The server uses pre-indexed [7] data to speed up random access and returns only the necessary information for the visualization back to the client. Hence, this approach becomes much more scalable even with large sized genomic data sets. We have implemented bedserver as a Python package (https://github.com/drio/bedserver) although we expect users will create their own sources and backends to interact with the specific details of their environments.

The source code of our library (and the original Cubism) has a decoupled interface that facilitates the extension of this library to new data sources. DNAism is data agnostic. As a result, users can create new sources to capture their specific backend peculiarities.

### Reproducibility

We consider in this section the two main aspects of reproducibility: first the ability of the software to generate the same results given the same input sets, and second the requirements for our users to install and use our software, that is the ability of new users to reproduce and exploit the capabilities we intend.

The main goals of our library are to visually encode data points that capture the value of some variable under study for a series of genomic locations and to display those values on a computer screen. This makes validation rather simple. We can inspect a small area of the genome and check the actual data points displayed against our input files. Once the interesting patterns and behavior are discovered in the datasets, the user can proceed to manually confirm the results by looking back at the raw data.

Since our contribution is released as a library, the user must write some minimal code to interact with the library. This additional user-provided code is small but requires a basic understanding of the technologies that are used within the web ecosystem: HTML, CSS and Javascript. In addition, since the library relies on D3js for rendering, an understanding of this technology is necessary [8]. That being said, the examples and interface documentation should help users to start using the library quickly. Once they are familiar with the examples, they can spend a bit of time exploring further into the details of how the code translates in pixel rendering, although that is not necessary to fully exploit the benefits of DNAism.

There is no installation required since this is a Javascript library. The library code is included within the web application that uses DNAism and the functionality is exposed via an object in the first level namespace object called DNAism.

## Results and discussion

The main function of DNAism is to expose the power of Horizon Charts while abstracting the inner details. Exposing the functionality as a library provides flexibility to the user to allow them to incorporate these visualization techniques within their projects. We believe that this technology is ideal for developing visualizations that will help the community to better understand their genomic datasets.

We are not aware of any other tools that use Horizon Charts to explore the genomic data.

The library is intended for exploring genomic data. It is ideal for aiding quality control on genomic datasets by visualizing different encoded metrics, typically in BED format.

In the future, we will be adding new sources to allow the users to load data from different types of backend services. We want to extend the library to make it easier to use, especially for the users that are not well-versed with web ecosystem.

## Conclusion

We introduce a powerful visualization technique previously used in the time series data domain. This visual tool facilitates the identification of similarities or abnormalities in patterns across multi-sample datasets. In addition, this approach helps to explore and visualize high density datasets more effectively, thereby helping the researchers to understand their data more easily.

Our library keeps the effective and elegant interface of the original, while allowing users to leverage its power for genomic data. By providing a library, we maintain flexibility regarding how researchers can use these resources. Users can build full applications or use the library within their existing ones.

The companion lightweight server will facilitate the exploration of large genomic datasets without affecting user experience, by using indexed datasets. Alternatively, users can create their own data sources to reflect the details of their own environments.

## Availability and requirements

**Project name:** DNAism.**Project home page:**http://drio.github.io/dnaism (main site).**Source code:**https://github.com/drio/dnaism.**Operating system(s):** Platform Independent.**Programming language:** JavaScript.**Other requirements:** Modern Browser.**License:** Apache 2.0.

## Additional file

Additional file 1
**Figure S1.** Ucsc genome browser displaying multiple tracks. (KB 106 PDF)

## References

[1] Few S. Time on the horizon. Visual Business Intelligence Newsletter. 2008. http://www.perceptualedge.com/articles/visual_business_intelligence/time_on_the_horizon.pdf.

[2] Saito T, Miyamura HN, Yamamoto M, Saito H, Hoshiya Y, Kaseda T (2005). Two-tone pseudo coloring: Compact visualization for one-dimensional data. Proceedings of the Proceedings of the 2005 IEEE Symposium on Information Visualization. INFOVIS ’05.

[3] Heer J, Kong N, Agrawala M. Sizing the horizon: the effects of chart size and layering on the graphical perception of time series visualizations. In: Proceedings of the SIGCHI Conference on Human Factors in Computing Systems. Association for Computing Machinery: 2009. p. 1303–1312.

[4] Wang J, Kong L, Gao G, Luo J (2013). A brief introduction to web-based genome browsers. Brief Bioinformatics.

[5] Kuhn RM, Haussler D, Kent WJ (2012). The UCSC genome browser and associated tools. Brief Bioinformatics.

[6] Bostock M, Ogievetsky V, Heer J (2011). D^3^ data-driven documents. IEEE Trans Vis Comput Graph..

[7] Li H (2011). Tabix: fast retrieval of sequence features from generic tab-delimited files. Bioinformatics.

[8] Wang R, Perez-Riverol Y, Hermjakob H, Vizcaíno JA (2015). Open source libraries and frameworks for biological data visualisation: A guide for developers. Proteomics.

