# Supplementary figures and images for: DNAism: exploring genomic datasets on the web with Horizon Charts
Source: BMC Bioinformatics. 2016 Jan 27;17:49. doi: 10.1186/s12859-016-0891-2 (PMC4729118; doi:10.1186/s12859-016-0891-2)

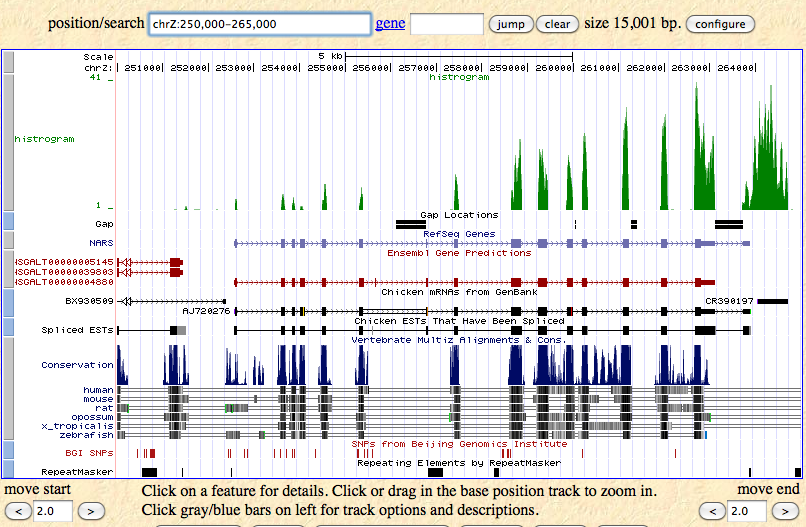

Supplement: Additional file 1 — Figure S1. Ucsc genome browser displaying multiple tracks. (KB 106 PDF) [file 12859_2016_891_MOESM1_ESM.png]
